# Brain regions differences in amyloid-**β** and gene expression in early APP/PS1 mice and identification of Npas4 as a key molecule in Alzheimer’s disease

**DOI:** 10.17305/bb.2024.10820

**Published:** 2024-12-01

**Authors:** Niya Wang, Zhong Zhao, Xiaoyan Wang, Xinzhang Chen, Fengwen Jiang, Yahong Tan, Wenli Chen, Qiang Meng

**Affiliations:** 1 Department of Neurology, The First People’s Hospital of Yunnan Province, The Affiliated Hospital of Kunming University of Science and Technology, Kunming, China; 2 Key Laboratory of Animal Models and Human Disease Mechanisms, Laboratory of Learning and Memory, Kunming Institute of Zoology, The Chinese Academy of Sciences, Kunming, China

**Keywords:** Alzheimer’s disease (AD), amyloid-β (Aβ), cerebellum, cortex, hippocampus, neuronal PAS domain protein 4 (Npas4)

## Abstract

Distinct brain regions are differentially affected during the various stages of Alzheimer’s disease (AD). While the hippocampus and cortex are known to play significant roles, the involvement of the cerebellum has received less attention. Understanding the changes in diverse brain regions is essential to unravel the neuropathological mechanism in early-stage AD. Our research aimed to explore and compare amyloid-β (Aβ) pathology and gene expression profiles across the hippocampus, cortex, and cerebellum in the early stages of the Amyloid Precursor Protein/Presenilin-1 (APP/PS1) mouse model. By seven months of age, significant Aβ plaque accumulation was observed in the hippocampus and cortex of APP/PS1 mice, while no such deposits were found in the cerebellum. Gene expression analysis revealed predominant effects on immune response pathways in the hippocampus and cortex. Even in the absence of Aβ deposition, notable gene expression changes were observed in the cerebellum of APP/PS1 mice. Intriguingly, neuronal PAS domain protein 4 (Npas4) expression was consistently downregulated across all brain regions, independent of Aβ plaque presence. Our findings reveal distinct transcriptomic alterations and Aβ pathology in select cerebral regions during the initial phase of AD. Notably, the diminished expression of the Npas4 across three brain regions implies that Npas4 could play a pivotal role in the early pathogenesis of AD.

## Introduction

Alzheimer’s disease (AD) is a progressive neurodegenerative disorder characterized by memory impairment, amyloid-β (Aβ) deposition, and neurofibrillary tangles (NFT) [1]. The hippocampus, an integral component of the limbic system situated between the thalamus and the medial temporal lobe, is essential for the storage and retrieval of long-term memory [2, 3]. Neuropathological changes in AD are thought to initially begin in the hippocampus and entorhinal cortex regions and gradually progress to the frontal, parietal, and temporal regions of the brain [4, 5]. Notably, the hippocampus is profoundly affected in AD [6, 7]. While the cerebral cortex provides the structural foundation for higher neural functions and its involvement in AD has garnered attention, the cerebellum—traditionally viewed as pivotal solely for autonomic motor control and learning—might also play a role in cognitive processes and memory. Nonetheless, cerebellar research in AD is nascent, and numerous aspects remain to be elucidated [8, 9].

Synaptic loss is the most salient pathological feature correlating with the cognitive deficits observed in AD. Accumulating evidence suggests that the pathophysiology of AD results from intricate interactions among pathological proteins, synaptic structures, and glial cells [10, 11]. Neuronal PAS domain protein 4 (Npas4) belongs to the basic helix–loop–helix (bHLH)-PAS protein family and is an activity-dependent transcription factor expressed in brain neurons. Npas4 has been associated with synaptic plasticity and memory formation, it plays a role in regulating the activity-dependent gene expression in neurons, which is important for long-term memory formation and synaptic function [12–14]. The expression changes of Npas4 in AD, especially in different brain regions, are still unclear.

Previous investigations, including our own, have documented Aβ deposition in the hippocampus and cortex of amyloid precursor protein/Presenilin-1 (APP/PS1) mice at the age of 6–7 months, with the cerebellum receiving comparatively less scrutiny [15, 16]. In this study, we assessed the Aβ plaque burden and APP expression in the hippocampus, cortex, and cerebellum of 7-month-old APP/PS1 mice. Our findings revealed significant Aβ plaque accumulation in the hippocampus and cortex, but no deposition in the cerebellum, notwithstanding an increase in APP protein expression across all three regions. Comprehensive transcriptome sequencing performed on hippocampal, cortical, and cerebellar tissues from APP/PS1 and wild-type (WT) mice illuminated differences and commonalities in gene expression across these brain regions between AD and WT. Particularly, the study spotlighted the gene “*Npas4*,” pivotal in learning and memory, as a potential key player in all three regions. Subsequent immunofluorescence and western blot experiments confirmed Npas4 protein expression and cellular localization by elucidating Aβ deposition patterns and gene expression in early-stage APP/PS1 mice across different brain regions and identifying *Npas4* as a key gene. The Npas4 expression is decreased and not associated with Aβ plaque deposition. This study offers novel insights and theoretical foundations for AD prevention and treatment strategies.

## Materials and methods

### Animal model

The male APP/PS1 transgenic mice (B6. Cg-Tag [APPswe, PSEN1dE9] 85Dbo/Mmjax, from the Model Animal Research Centre of Nanjing University, Nanjing, China) and their negative littermates were used. The mice were housed in ventilated cages with 4–5 littermates and had unlimited access to water and food. They were kept in a temperature-controlled environment with a 12-h light/dark cycle.

### RNA extraction, library construction, and sequencing

Mice were anesthetized with pentobarbital sodium (80 mg/kg, intraperitoneally) and subsequently transcardially perfused with phosphate-buffered saline (PBS). Brains were extracted, and the hippocampus, cortex, and cerebellum were isolated and stored at –80 ^∘^C in RNAlater (ThermoFisher). For each experimental group, six mice were utilized. RNA sequencing was conducted by Novogene (Beijing, China). Total RNA was isolated using TRIzol reagent, and sequencing libraries were prepared with the NEBNext Ultra RNA Library Prep Kit for Illumina (NEB, USA) following the provided guidelines, including the addition of index codes for sample identification. Libraries were purified using the AMPure XP system and their quality was evaluated on an Agilent Bioanalyzer 2100 system. Clustering of the indexed samples was carried out on a cBot Cluster Generation System with the HiSeq 4000 PE Cluster Kit (Illumina) as per the manufacturer’s instructions. Sequencing was performed on an Illumina HiSeq 4000 platform, producing 150 bp paired-end reads.

### RNA-seq data analysis

Initially, we implemented data quality control procedures to ensure clean reads for further analysis. The raw data was filtered, sequencing error rates were evaluated, and the GC content distribution was inspected. Subsequently, an index of the reference genome was constructed using Hisat2 (v2.0.5) and the paired-end clean reads were aligned with the reference genome using the same software. Gene expression levels were estimated by calculating the fragments per kilobase of exon per million mapped fragments (FPKM) with FeatureCounts (1.5.0-p3). We determined correlation coefficients for both intra-group and inter-group samples based on FPKM values of all genes within each sample. In preparation for differential gene expression analysis, the read counts for each library were normalized using the edgeR program package and a scaling normalized factor was applied. We used the edgeR R package (version 3.22.5) to perform differential expression analysis between two conditions, adjusting *P* values according to the Benjamini & Hochberg method. We considered genes with an adjusted *P* value (*p*adj) of less than 0.05 and an absolute log2 fold change greater than 0.585 to be significantly differentially expressed. Finally, gene enrichment and functional annotation analyses were conducted based on the Gene Ontology (GO) and KEGG databases. GO terms and KEGG pathways with a corrected *P* value less than 0.05 were considered significantly enriched by differentially expressed genes (DEGs).

### Quantitative real-time PCR (qRT-PCR)

RNA was extracted using the Eastep™ Total RNA Super extraction kit, and mRNA was reverse transcribed into cDNA utilizing GoScript Reverse Transcription Mix (Promega, A28). The qRT-PCR reaction mix, totaling 20 µL, comprised 10 µL of 2× SYBR qPCR MasterMix (GlpBio, GK10002), 0.4 µL of each 10 µM primer (Npas4: TGACAGAAAGCTCAGCAGA, GTTGCTCTGGGAAGGTTTGG; GAPDH: CAGTGGCAAAGTGGAGATTGTTG, TCGCTCCTGGAAGATGGTGAT), 1 µL of template cDNA, and the balance made up with 8.2 µL of ddH_2_O. Amplification was conducted on a LightCycler480 (Roche, Germany), with an initial denaturation at 95 ^∘^C for 30 s, followed by 40 cycles at 95 ^∘^C for 15 s and 60 ^∘^C for 30 s. Each sample was assayed in triplicate, alongside a no-template control for each targeted gene. Quantitative data were analyzed using the 2^−ΔΔCt^ method.

### Immunofluorescence

Mice were anesthetized using pentobarbital sodium (80 mg/kg, intraperitoneally) and successively perfused transcardially with PBS followed by 4% paraformaldehyde (PFA). Subsequently, brains were extracted, post-fixed in 4% PFA at 4 ^∘^C overnight, and dehydrated in 30% sucrose/PBS solution at 4 ^∘^C for 48 h. Coronal brain sections of 30 µm thickness were prepared using a freezing microtome (RWD, China). For antigen retrieval, sections were heated in a citrate antigen retrieval solution at 95 ^∘^C for 5 min. Afterward, sections were rinsed thrice for 10 min with 0.01 M PBS and blocked with a solution containing 5% bovine serum albumin and 0.3% Triton X-100 for 1 h. The sections were then incubated with primary antibodies Npas4 (1:1000, N408/79, NeuroMab, mouse), NeuN (1:1000, #24307, Cell Signaling, rabbit), and Aβ (D54D2, 1:1000, 51374, Cell Signaling) overnight at 4 ^∘^C. Following three washes in 0.01 M PBS, sections were incubated with fluorescently labeled secondary antibodies (1:1000, Proteintech Group) for two hours at room temperature. After three PBS washes, the sections were mounted with a DAPI-containing medium and coverslipped. Control sections omitted the primary antibody, resulting in no detectable specific labeling. Imaging was performed using a Nikon A1 confocal microscope.

### Western blot

The experimental methodology aligns with our previous work. Homogenization of mouse brain tissue was performed using RIPA Lysis Buffer (Beyotime Biotech), supplemented with PMSF (Selleck) and a protease inhibitor cocktail (GlpBio). Protein concentrations were determined via the BCA assay. Subsequently, samples were separated on a 7.5% SDS-PAGE (PAGE Gel Fast Preparation Kit, Epizyme) and transferred to PVDF membranes (Millipore). Blocking was conducted for 1 h at room temperature using TBST (0.9% NaCl, 10-mM Tris, 0.1% Tween-20, pH 7.4) containing 5% BSA, on an orbital shaker. Primary antibodies were applied overnight at 4 ^∘^C, targeting APP (1:5000, ab32136), Npas4 (1:1000, SAB2501493, Sigma-Aldrich; 1:1000, N408/79, NeuroMab), and α-tubulin (1:10000, ab176560, Abcam). Following three 10-min washes in TBST, membranes were incubated with an HRP-conjugated secondary antibody (1:10000, Proteintech Group) for 2 h at room temperature. Immunoreactivity was visualized using a Gel Imaging System (Tannon 5200 Multi) and analyzed with Image J software.

### Ethical statement

All experimental procedures were approved by the Institutional Animal Care and Use Committee of Kunming Institute of Zoology, the Chinese Academy of Sciences, Kunming 650223, China (SMKX-2015019, IACUC-RE-2023-11-001).

### Statistical analysis

Statistical analyses were carried out using GraphPad Prism 9. Unpaired Student’s *t*-tests were used to compare data between groups, and one-way ANOVA was used to assess multiple groups, followed by post hoc multiple comparison tests. The data was displayed as mean ± standard error of the mean (SEM), and a *P* value less than 0.05 was considered statistically significant.

**Figure 1.**
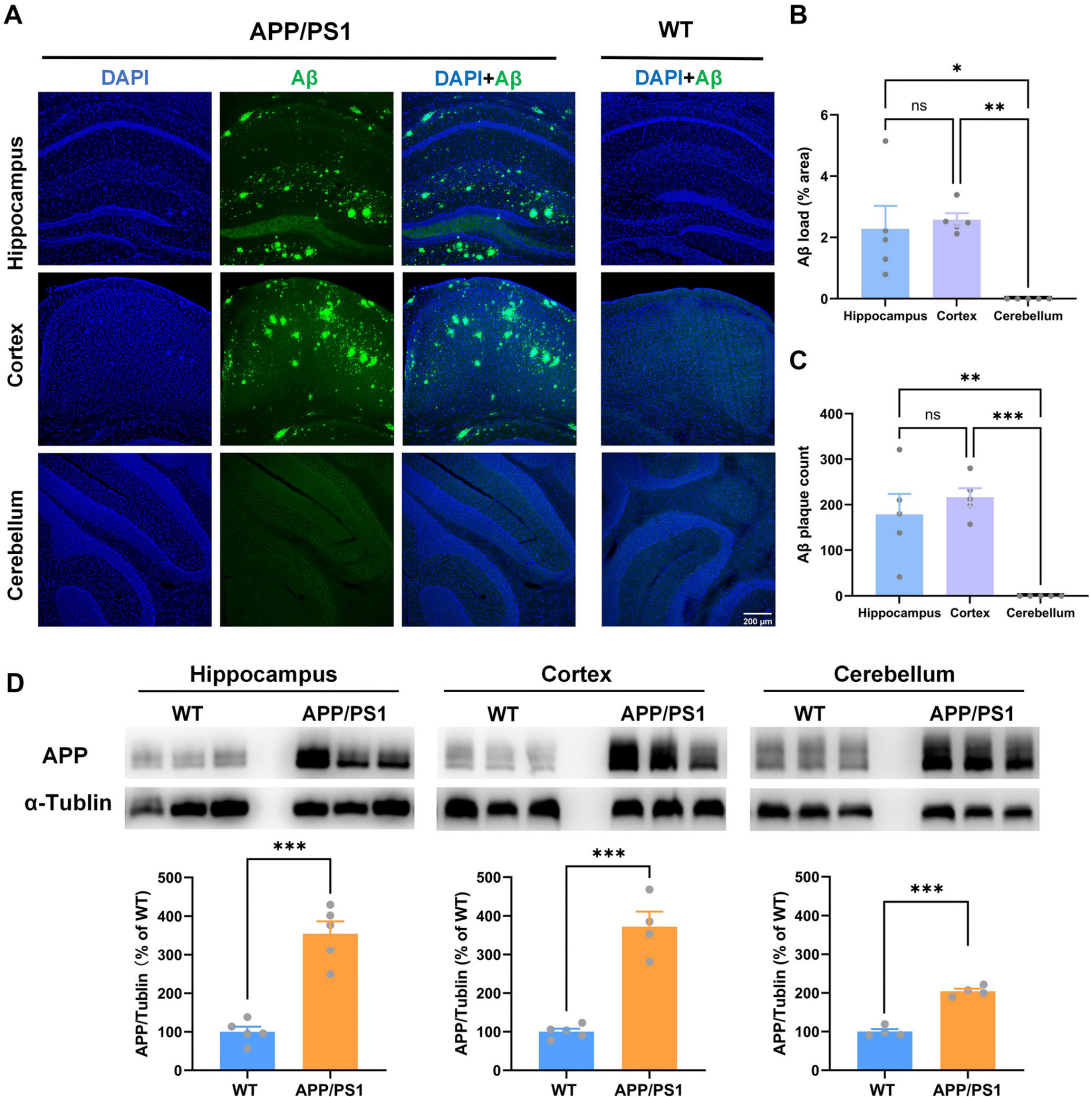
**Aβ plaque load and APP protein expression in the hippocampus, cortex, and cerebellum in APP/PS1 mice at seven months.** (A) Representative images of immunostaining for Aβ plaque in the hippocampus, cortex and cerebellum in APP/PS1 and WT mice at 7-month old; (B) Aβ plaque load (% area) (one-way ANOVA: *F*_(2, 12)_ ═ 9.536, *P* ═ 0.003; Hip vs Cor, *P* ═ 0.889; Hip vs Ceb, *P ═ 0.011; Cor vs Ceb, **P ═ 0.005) and (C) Aβ plaque count (one-way ANOVA: *F*_(2, 12)_ ═ 16.03, *P* < 0.001; Hip vs Cor, *P* ═ 0.637; Hip vs Ceb, **P ═ 0.002; Cor vs Ceb, ***P < 0.001) were detected in the hippocampus and cortex of APP/PS1 mice, but no Aβ plaque was observed in either the cerebellum of APP/PS1 mice or in these regions of WT mice, at 7-month old; (D) APP protein was expressed consistently in these brain regions with higher levels in APP/PS1 mice than in WT mice (*t-*test: Hippocampus [*t* ═ 7.242, df ═ 8, ***P < 0.001]; cortex [*t* ═ 7.748, df ═7, ***P < 0.001]; cerebellum [*t* ═ 10.97, df ═ 6, ***P < 0.001]). Hip: Hippocampus; Cor: Cortex; Ceb: Cerebellum; WT: Wild-type; APP: Amyloid precursor protein; APP/PS1: Amyloid precursor protein/Presenilin-1; DEG: Differentially expressed gene.

**Figure 2.**
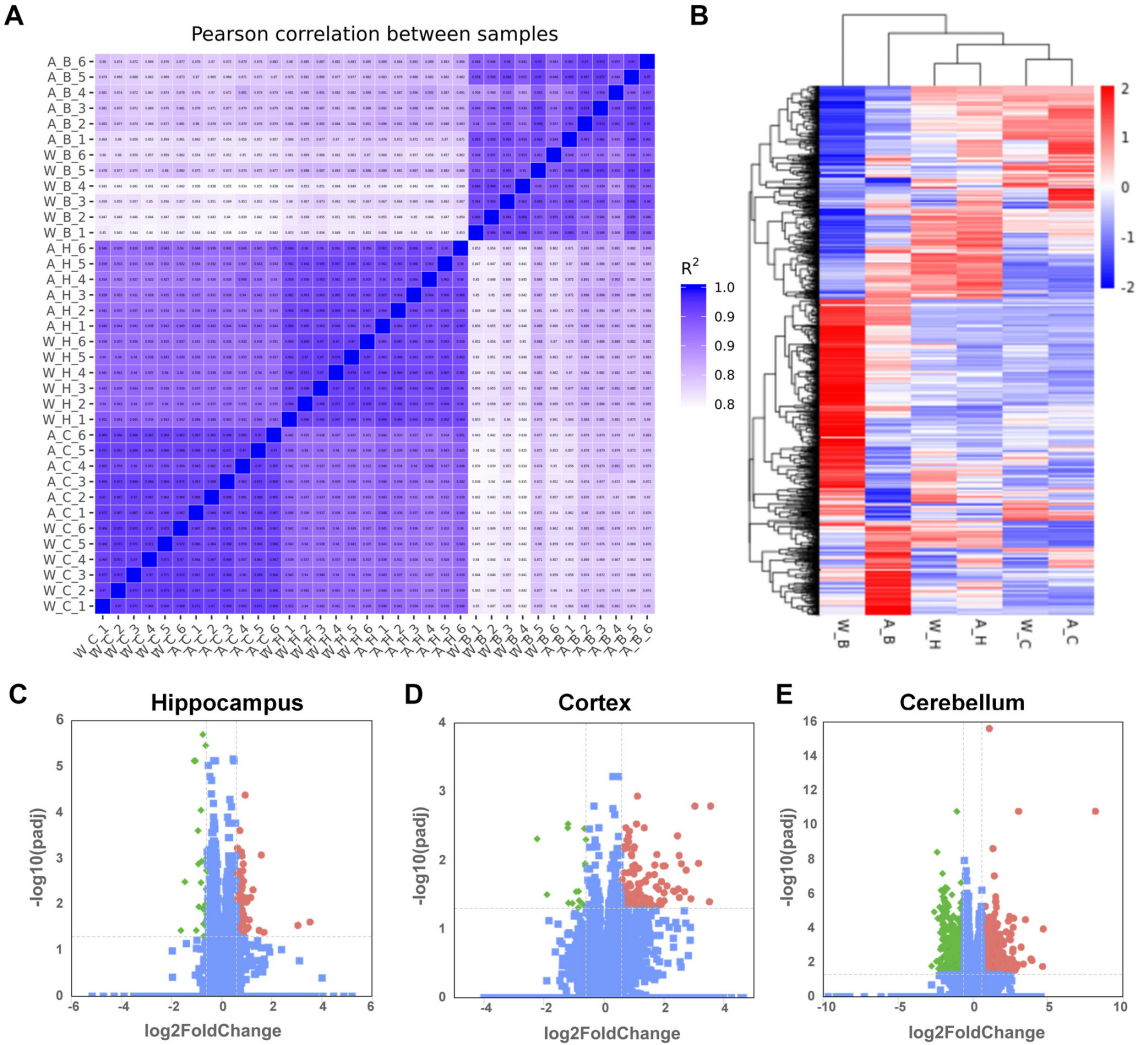
**The expression of transcripts of the three brain regions of APP/PS1 mice and WT mice.** (A) Pearson correlation coefficients for comparisons among all samples show the highest similarity between the replicates of each group; (B) Heat map of significantly DEGs (*P* < 0.05), with red indicating upregulation and blue indicating downregulation; (C–E) Volcano plot of gene expression with significantly increased (red) or decreased (green) expression (*P* < 0.05, |log2 fold change| > 0.585) in hippocampus, cortex, and cerebellum compared with the corresponding controls. (W_H: Hippocampus of WT mice group, A_H: Hippocampus of APP/PS1 mice group, W_C: Cortex of WT mice group, A_C: Cortex of APP/PS1 mice group, W_B: Cerebellum of WT mice group, A_B: Cerebellum of APP/PS1 mice group). WT: Wild-type; APP/PS1: Amyloid precursor protein/Presenilin-1; DEG: Differentially expressed gene.

## Results

### A**β** plaque deposited in the hippocampus and cortex but not in the cerebellum in APP/PS1 mice at 7-month old

Previous studies have established that cognitive dysfunction typically manifests in 6–7-month-old APP/PS1 mice. Our study confirmed this finding and also revealed Aβ plaques in the hippocampus, cortex, and cerebellum of 7-month-old APP/PS1 and WT mice through immunostaining. Notably, we observed a significant buildup of Aβ plaques in the hippocampus and cortex of APP/PS1 mice, while the cerebellum showed minimal Aβ accumulation (Figure 1A–1C). Western blotting analysis further showed increased levels of APP in all three brain regions of APP/PS1 mice compared to WT mice (Figure 1D). Intriguingly, despite the absence of Aβ deposition in the cerebellum of 7-month-old APP/PS1 mice, a stark contrast to the hippocampus and cortex, the increase in APP expression was consistent across these regions. This prompts further investigation into potential pathological changes occurring in the cerebellum of APP/PS1 mice, particularly in comparison with the hippocampus and cortex, to elucidate any differential impacts.

### Transcriptome comparisons of three brain regions of APP/PS1 mice against WT counterparts

To investigate the differential gene expression patterns between APP/PS1 and WT mice across three brain regions, transcriptome sequencing was performed on the hippocampus, cortex, and cerebellum tissues from APP/PS1 and WT mice. An impressive 1.66 billion raw reads were generated from total RNA of 36 samples using the IlluminaHiSeq 4000 platform. Post-quality control, an average of approximately 45.3 million clean reads per sample were mapped to the reference genome. The fidelity of the samples within and between groups was affirmed by a high Pearson correlation coefficient, ranging between 0.77 and 0.98 (*R* > 0.75), indicating high repeatability among the samples in the group, as visualized in the generated heat maps (Figure 2A). Differentially expressed mRNA transcripts were analyzed through hierarchical clustering, revealing distinct patterns (Figure 2B). Notably, the cerebellum exhibited a greater number of DEGs compared to other regions. Increased gene expression was noted in 66, 110, and 974 genes within the hippocampus, cortex, and cerebellum of APP/PS1 mice, respectively, whereas 30, 16, and 818 genes demonstrated decreased expression (Figure 2C–2E). Interestingly, among the cerebellum DEGs, several categories including “antisense,” “processed pseudogene,” and “TEC (to be experimentally confirmed)” genes were predominant.

**Figure 3.**
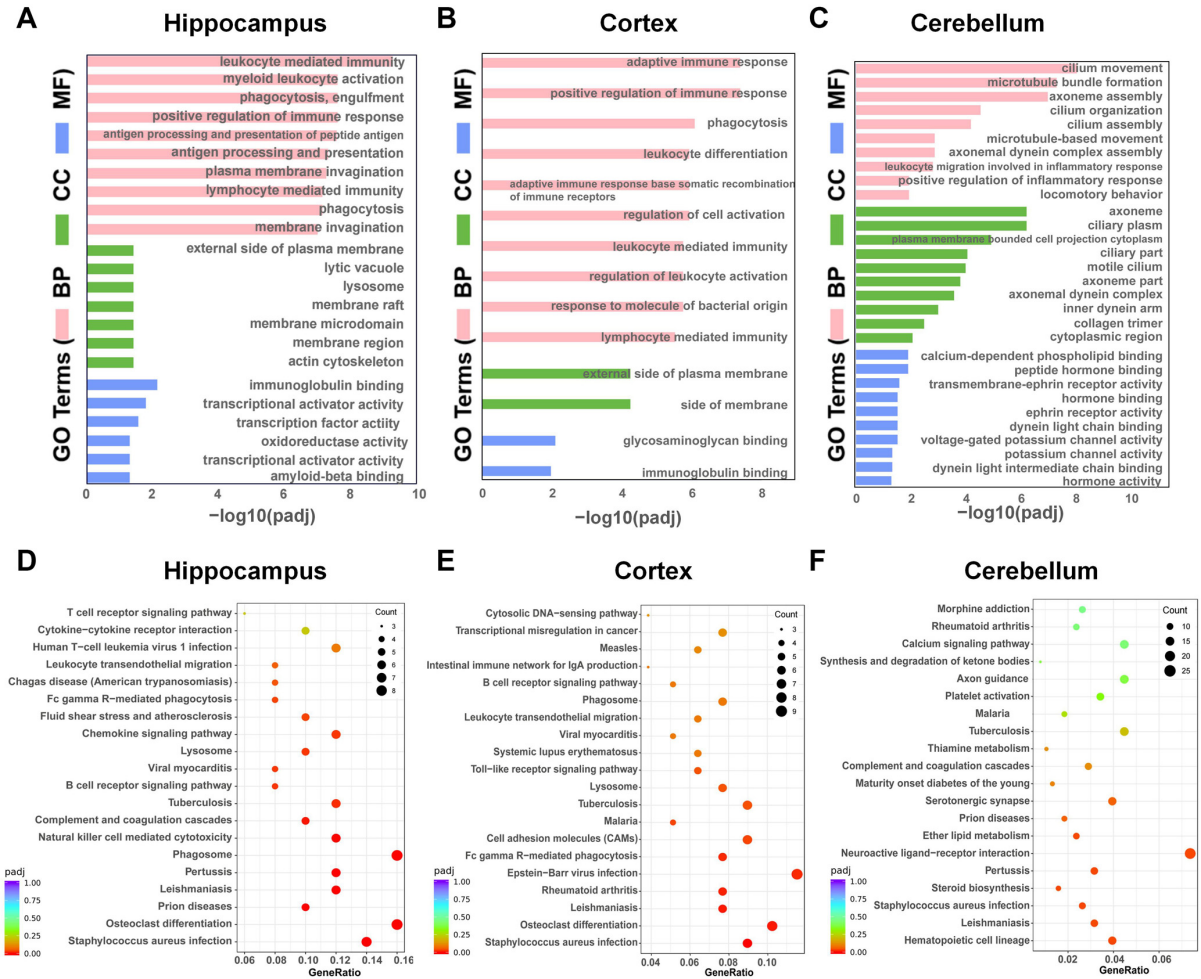
**Functional enrichment analysis of DEGs for three brain regions.** (A–C) GO function enrichment analysis of the DEGs in hippocampus (A), cortex (B) and cerebellum (C) between APP/PS1 and WT mice. (D–F) KEGG pathway enrichment analysis of DEGs in hippocampus (D), cortex (E), and cerebellum (F) between APP/PS1 and WT mice. WT: Wild-type; GO: Gene ontology; DEGs: Differentially expressed genes; APP/PS1: Amyloid precursor protein/Presenilin-1; KEGG: Kyoto Encyclopedia of Genes and Genomes.

We conducted functional enrichment analyses using the GO and KEGG databases to elucidate the molecular mechanisms across various brain regions. Significant enrichment was observed for 410 GO terms in the hippocampus, 303 in the cortex, and 71 in the cerebellum, when comparing 7-month-old APP/PS1 mice with WT littermates (*q* < 0.05). Notably, Figure 3A presents the top ten GO terms for the hippocampus across the biological process (BP), cellular component (CC), and molecular function (MF) categories. Enriched BPs included leukocyte-mediated immunity and myeloid leukocyte activation; CCs included the external side of the plasma membrane and lytic vacuole; MFs included immunoglobulin binding and oxidoreductase activity. Figure 3B outlines the cortex’s top ten GO terms, with BPs such as adaptive immune response; CCs as the external side of the plasma membrane; and MFs like glycosaminoglycan binding. For the cerebellum, Figure 3C reveals enrichment in ciliary motion (BP), axoneme (CC), and calcium-dependent phospholipid binding (MF). The term “positive regulation of immune response” was enriched in all three brain regions, suggesting inflammation in AD. There are more similar enrichment pathways in the hippocampus and cortex. The hippocampus and cortex also showed similar enriched pathways, including immune response and phagocytosis, while the main pathways in the cerebellum were cilia and microtubule movement and assembly.

KEGG pathway analysis indicated significant enrichment in 16 pathways in the hippocampus, 11 in the cortex, and 7 in the cerebellum (Figure 3D–3F). Notably, *Staphylococcus aureus* infection and osteoclast differentiation pathways were prevalent in the hippocampus and cortex, while neuroactive ligand–receptor interaction and ether lipid metabolism pathways were distinguished in the cerebellum. Predominantly, genes in these pathways were upregulated and closely associated with immune response functions.

### The key gene *Npas4* was downregulated in all three brain regions

**Figure 4.**
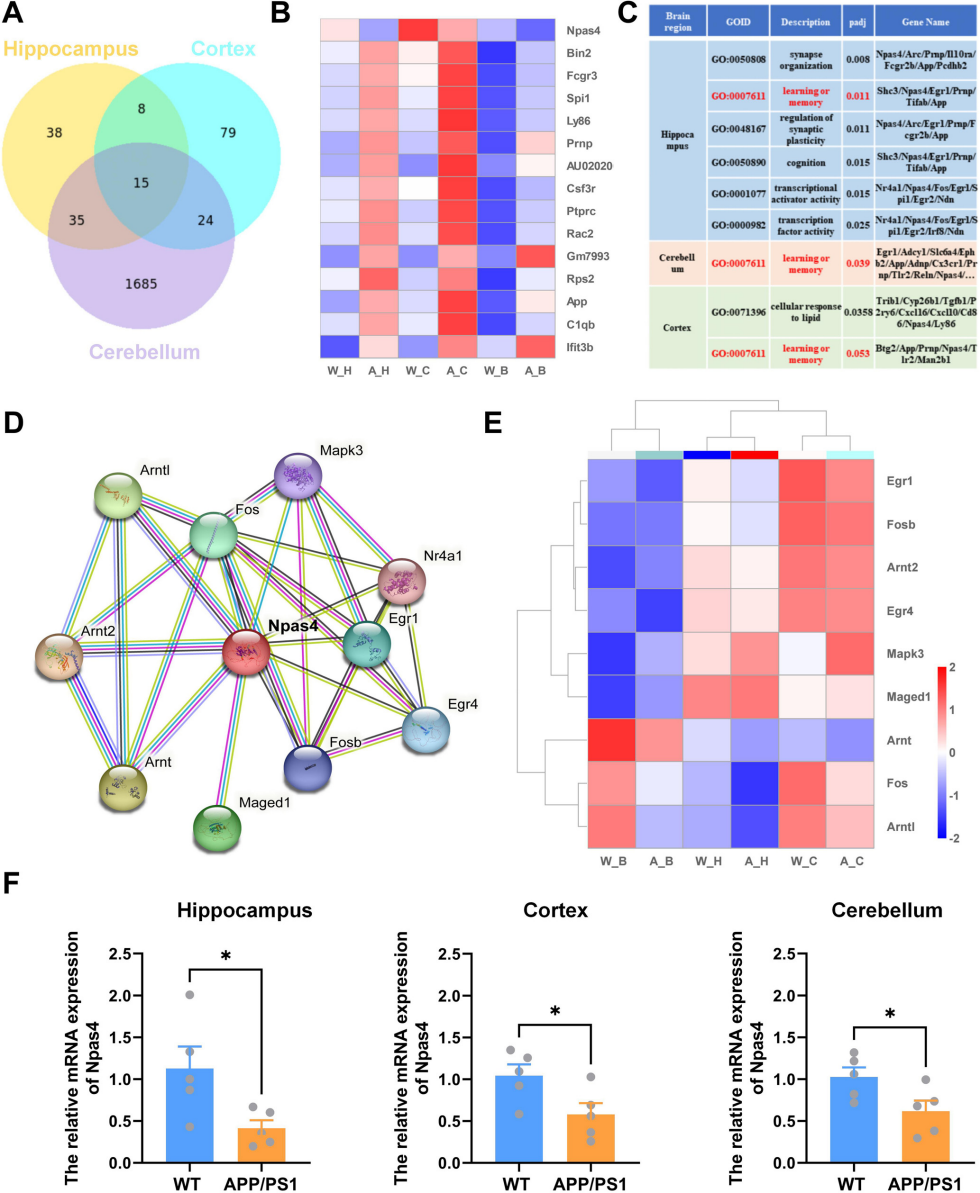
**The overlap of DEGs across three different brain regions and the *Npsa4* gene network phenotype.** (A) Venn diagrams illustrating the overlap of DEGs across three different brain regions from APP/PS1 mice and WT mice; (B) The overlapped 15 regulated genes were listed along with their expression changes; (C) The *Npas4* gene was involved in the GO terms for “learning or memory” in the hippocampus, cortex, and cerebellum from APP/PS1 mice versus WT mice; (D) A network of genes that interact with *Npas4* in “STRING 12.0”; (E) Heat cluster patterns showing mRNA transcripts of the *Npas4* network in the hippocampus, cortex, and cerebellum from APP/PS1 mice versus WT mice; (F) The validation of *Npas4* mRNAs in the hippocampus (*t-*test: *t* ═ 2.553, df ═ 8, **P ═ 0.034), cortex (*t-*test: *t* ═ 2.412, df ═ 8, *P ═ 0.042) and cerebellum (*t-*test: *t* ═ 2.407, df ═ 8, *P ═ 0.043) from APP/PS1 mice vs WT mice. Npas4: Neuronal PAS domain protein 4; WT: Wild-type; GO: Gene ontology; DEGs: Differentially expressed genes; APP/PS1: Amyloid precursor protein/Presenilin-1.

We assessed the shared significant DEGs among three brain regions in APP/PS1 mice compared to WT counterparts, using a Venn diagram to depict the 15 common genes (Figure 4A). Of these, 14 genes, including *Bin2*, *Fcgr3*, *Spi1*, *Ly86*, *Prnp*, *AU020206*, *Csf3r*, *Ptprc*, *Rac2*, *Gm7993*, *Rps2*, *App*, *C1qb*, and *Ifit3b*, were consistently upregulated across the three regions in APP/PS1 mice (Figure 4B). Notably, the upregulation of the APP and *Prnp* genes aligns with the genetic profile of these transgenic mice and our prior observations of heightened APP protein levels (Figure 1). The remaining upregulated genes predominantly relate to inflammatory processes. Conversely, *Npas4* was the sole gene found to be downregulated across all brain regions in 7-month-old APP/PS1 mice. *Npas4* is an immediate early gene (IEG) and activity-dependent transcription factor in neuronal tissue, critical for encoding experiences to forge long-term memories. GO analysis highlighted several *Npas4*-involved pathways, notably synaptic organization (GO:0050808), learning or memory (GO:0007611), and regulation of synaptic plasticity (GO:0048167), the “learning or memory” pathway was enriched in all three brain regions, underpinning *Npas4*’s linkage to cognitive dysfunction (Figure 4C). Protein network interactions with Npas4 were investigated using the STRING database (Figure 4D), with heat cluster patterns demonstrating mRNA transcript variation of the *Npas4*-associated network in the hippocampus, cortex, and cerebellum of APP/PS1 vs WT mice (Figure 4E). To validate our findings, we used quantitative RT-PCR and confirmed reduced expression of *Npas4* in all three regions, with a significant decrease in the hippocampus and a slight decrease in the cortex and cerebellum of APP/PS1 mice compared to WT (Figure 4F).

### Npas4 protein expression was decreased in all three brain regions

**Figure 5.**
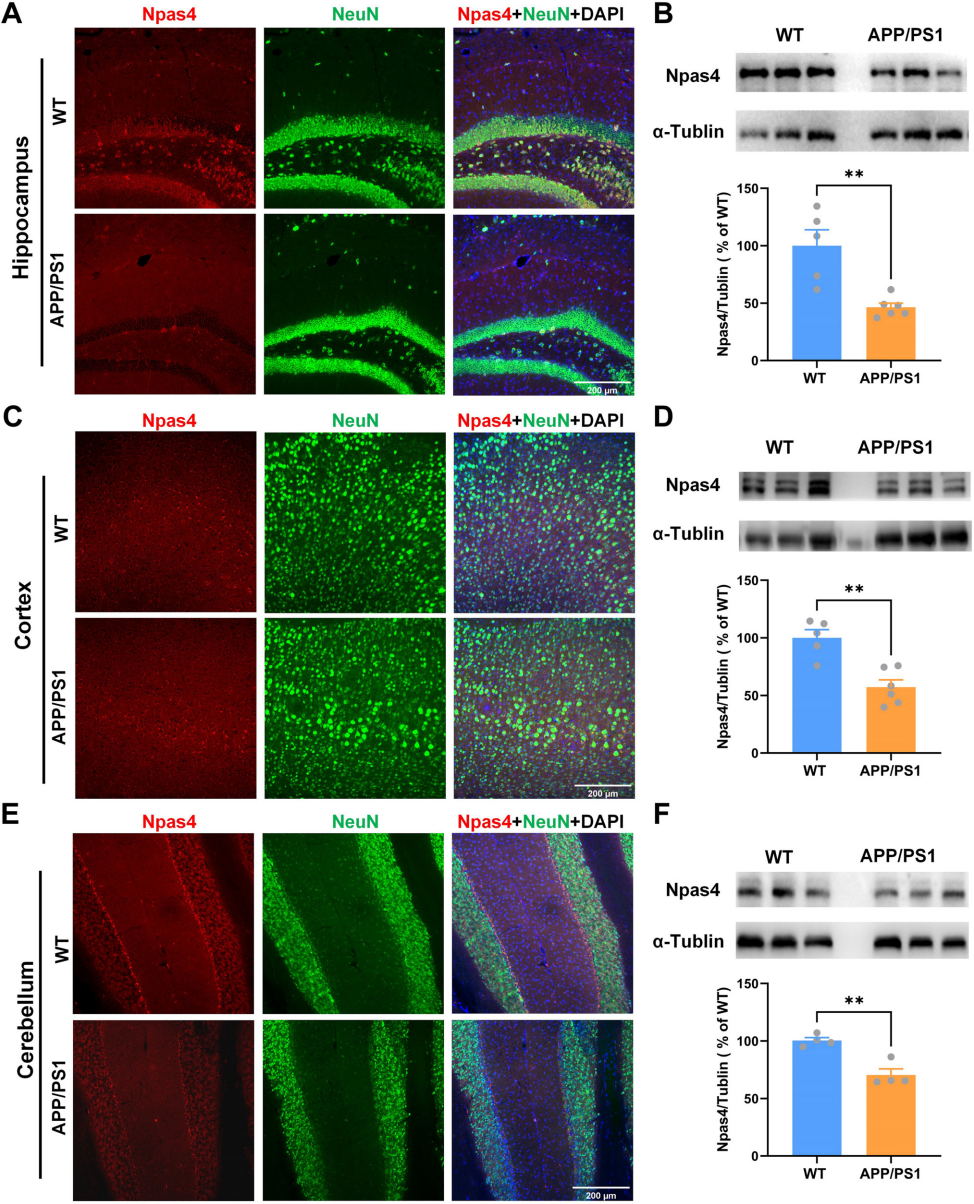
**The expression and localization of Npas4 protein in these three brain regions.** (A) Representative immunostaining images for Npas4 (red) and NeuN (green) in the hippocampus; (B) Protein expression of Npas4 (*t-*test: *t* ═ 4.083, df ═ 9, **P ═ 0.003) was significant reduced in the hippocampus in APP/PS1 mice compared to WT mice; (C) Representative immunostaining images for Npas4 (red) and NeuN (green) in the cortex; (D) Protein expression of Npas4 was significant reduced in the cortex in APP/PS1 mice compared to WT mice (*t-*test: *t* ═ 4.534, df= 9, *P* ═ 0.001); (E) Representative immunostaining images for Npas4 (red) and NeuN (green) in the cerebellum; (F) Protein expression of Npas4 was significant reduced in the cerebellum in APP/PS1 mice compared to WT mice (*t-*test: *t* ═ 5.109, df ═ 6, **P ═ 0.002). Npas4: Neuronal PAS Domain protein 4; WT: Wild-type; APP/PS1: Amyloid precursor protein/Presenilin-1.

The protein expression and cellular localization of Npas4 were further investigated in the hippocampus, cortex, and cerebellum. Immunofluorescence staining, along with double staining of Npas4 (red) and NeuN (green), revealed that Npas4 was predominantly expressed in neurons. In the hippocampus, immunofluorescence staining demonstrated a significant decrease in Npas4 expression in APP/PS1 mice compared to WT mice, particularly in the DG region (Figure 5A). This reduction in Npas4 expression was further confirmed by Western blot analysis, showing a notable decrease in Npas4 protein levels in APP/PS1 mice (Figure 5B). In the cortex, the npas4 expression was also decreased (Figure 5C and 5D). Furthermore, the protein expression of Npas4 was significantly decreased in the cerebellum of APP/PS1 mice (Figure 5E and 5F). Though Npas4 is primarily present in the hippocampus, it can also be found in other areas, such as the amygdala, fornix, and cingulate gyrus [17–19]. Our experiments also revealed the expression of Npas4 in the cerebellum of APP/PS1 mice, with the most notable reduction observed in the hippocampus when compared to WT mice. In our experiments, we found that Npas4 expression occurred in the cerebellum of APP/PS1 mice, with the most pronounced reduction noted in the hippocampus when compared to WT mice. Aβ plaque deposition did not show a correlation with Npas4 expression.

## Discussion

Historically, the cerebellum has been viewed as crucial for the control of autonomic motor functions and motor learning; however, emerging studies implicate its role in certain cognitive and mnemonic processes [20–22]. Earlier autopsy findings posited that Aβ plaques initially manifest in the cerebral cortex and, with the advancement of the disease, extend to subcortical areas, ultimately reaching the cerebellum in the terminal phase [23, 24]. Our study revealed significant Aβ plaque accumulation in the hippocampus and cortex but no presence in the cerebellum of 7-month-old APP/PS1 and WT mice. These observations align with those by Yu et al. [25], who reported an absence of Aβ plaques in the cerebellum of 6-month-old APP/PS1 mice, noting their emergence only at nine months. We noted a pronounced increase in APP protein expression across the hippocampus, cortex, and cerebellum in 7-month-old APP/PS1 mice compared to WT mice, an effect likely attributable to the genetic alterations in the transgenic models. Nonetheless, the delayed deposition of Aβ plaques within the cerebellum remains elusive and warrants further investigation.

In the analysis of the transcriptomic profiles of three brain regions from 7-month-old APP/PS1 mice compared to WT mice, it was surprising to discover a greater number of DEGs in the cerebellar region. Further analysis revealed that most of these different genes are “antisense”, “processed pseudogene”, “TEC (to be experimentally confirmed)” genes. The function of these genes is unknown, and their role in AD is even less clear. Consequently, the cerebellum had the fewest significantly enriched pathways identified by GO and KEGG enrichment analyses. In contrast, the hippocampus exhibited the most enriched pathways, followed by the cortex. Extensive research into transcriptomic changes in AD patients and animal models has identified numerous network disruptions indicative of diverse pathway alterations. These include, but are not limited to, immune function, synaptic transmission and plasticity, and glucose and lipid metabolism [16, 26–31]. Our study indicates that, in the early stages of AD, the DEGs in the hippocampus and cortex are chiefly enriched in pathways related to immune response and function as well as transcription factor activity. AD involves chronic inflammation and immune system dysregulation in the brain. The significance of the inflammatory response in AD pathogenesis is increasingly recognized, with evidence from preclinical, genetic, and biological studies linking immune system activation to AD pathology [32, 33]. Aβ is known to activate microglia and astrocytes, triggering an immune response, neuroinflammation driven by immune cells like microglia and astrocytes, which plays a significant role in the disease’s progression [34, 35]. The fewer enriched immune pathways in the cerebellum, compared to the hippocampus and cortex, may indeed be related to the lack of large Aβ deposits in this area. The immune response, including microglial activation and the inflammatory cascade, is closely tied to the presence of Aβ plaques and tau pathology. Therefore, brain regions with lower levels of Aβ may exhibit a reduced immune response, while the cerebellum may have fewer immune pathways activated in response to Aβ deposition, it may also play a role in the overall neuroinflammatory process in AD. Few pathways in the cerebellum were enriched, predominantly those involving microtubule bundle formation and axoneme assembly; this may be related to the structure and function of synapses, which needs further study.

In an investigation of DEGs across three brain regions, *Npas4* emerged as the sole gene consistently downregulated in all regions of 7-month-old APP/PS1 mice. This alteration in gene expression was corroborated through immunofluorescence and western blot analyses, which confirmed a reduction in Npas4 protein levels in these mice. Npas4, a transcriptional regulator pivotal for synaptic function, influences the expression of activity-dependent transcription factors, such as BDNF, Narp, and Kcna1 [36]. It has been reported that Npas4 may affect the function of excitatory and inhibitory neurons and memory formation [37–40]. Ramamoorthi et al. [41] demonstrated that *Npas4* deficits in both conditional *CA3* knockout and whole-brain knockout mice led to context memory impairments, which were ameliorated by restoring Npas4 expression. Npas4 has been shown to modulate MF-CA3 synapse numbers and synaptic plasticity by directing Plk2 kinase expression [42]. Rein et al. [43] found that a mouse model of autism with a mutation in the human *16p11.2* gene locus had reduced *Npas4* expression, and that synaptic and behavioral defect could be reversed by restoring *Npas4* expression. Recent studies have reported the regulation of Npas4 in anxious behavior [44, 45] and light rhythm regulation [46]. Genome-wide gene expression analysis found that *Npas4* gene was associated with NFTs, especially in Braak (NFTs) phase I-II significantly decreased [47]. Furthermore, Fan et al. [48] found that *Npas4* overexpression could induce autophagy and foster the clearance of endogenous tau protein in rat primary cortical neurons. 

Collectively, our findings and extant literature indicate that *Npas4* may be critically implicated in the cognitive deficits associated with AD. However, the underlying mechanisms remain elusive, necessitating further investigation into how *Npas4* regulates the neural circuitry abnormalities present in this condition. For example, the functional role of *Npas4* in neuronal health and AD pathology will be investigated in animal models through gene manipulation (such as knockdown or overexpression studies) to explore the effects of *Npas4* regulation on cognitive function and synaptic integrity in APP/PS1 mice, develop small molecule or gene manipulation to modulate *Npas4* and test its efficacy in preclinical AD models, and to explore the combination therapy of Aβ deposition and *Npas4* pathway to improve the therapeutic effect. Our findings confirm the pathology AD, namely, that certain brain regions are more susceptible to Aβ accumulation, leading to a better understanding of regional susceptibility to AD. Identifying the expression patterns of these genes in different brain regions could help reveal new molecular mechanisms driving AD pathology and potential biomarkers for early diagnosis. Considering *Npas4’s* role in regulating synaptic function and neuroprotection, it holds great promise as a therapeutic target. Modulating its activity may improve synaptic dysfunction and cognitive decline in AD patients.

## Conclusion

In conclusion, Aβ deposits were observed in the hippocampus and cortex but not in the cerebellum of 7-month-old APP/PS1 mouse models. Transcriptome sequencing revealed that immune response pathways in the hippocampus and cortex of APP/PS1 mice diverged significantly from those in WT mice of the same age. Despite the absence of Aβ deposition in the cerebellum at this age, notable transcriptomic alterations were detected. Further research is imperative to elucidate AD pathology in the cerebellum, the implications of cerebellar changes on brain connectivity, and the impact of such changes on cognitive function in AD and related disorders. Our research has pinpointed *Npas4* as a pivotal gene exhibiting differential expression across the three brain regions, and notably, its expression is not linked to Aβ plaque deposition. Recognized for its role in synaptic transmission and memory, the specific mechanism of *Npas4* in AD remains to be elucidated and could represent a critical target for understanding pathogenesis and devising treatment strategies. Improving our understanding of neurosynaptic changes during AD and aging is vital for identifying precise therapeutic targets and creating more effective treatments.

## Data Availability

The data supporting the findings of this study are available on request from the corresponding author. RNAseq raw data would be accessible in a data bank after paper published.
